# Epidemiology and genotypic diversity of human metapneumovirus in paediatric patients with acute respiratory infection in Beijing, China

**DOI:** 10.1186/s12985-021-01508-0

**Published:** 2021-02-18

**Authors:** Chao Wang, Tianli Wei, Fenlian Ma, Hao Wang, Jianqiang Guo, Aijun Chen, Yiman Huang, Zhiping Xie, Lishu Zheng

**Affiliations:** 1 NHC Key Laboratory of Medical Virology and Viral Diseases, National Institute for Viral Disease Control and Prevention, China CDC, Beijing, China; 2 Department of Pediatrics, Beijing Friendship Hospital, Capital Medical University, Beijing, China; 3 Center for Biosafety Mega-Science, Chinese Academy of Sciences, Beijing, China

**Keywords:** Human metapneumovirus, Epidemiology, Genetic diversity, Pediatric, Respiratory tract infection

## Abstract

**Background:**

Acute respiratory tract infections (ARTIs) causes high amounts of morbidity and mortality worldwide every year. Human metapneumovirus (HMPV) is a major pathogen of ARTIs in children. In this study, we aimed to investigate the epidemiology and genotypic diversity of HMPV in children hospitalized with ARTIs in Beijing, China.

**Methods:**

Hospitalized children aged < 14 years with ARTIs were enrolled from April 2017 to March 2018; nasopharyngeal aspirates were collected and subjected to real-time polymerase chain reaction tests for HMPV. HMPV-positive samples were genotyped based on a partial N gene. Whole genome sequences were determined for samples with high viral loads.

**Results:**

4.08% (52/1276) enrolled paediatric patients were identified as having HMPV infection. The epidemic season is winter and early spring, children aged ≤ 4 years were more susceptible to HMPV infection (47/52, 90.38%). The co-infection rate were 36.54% (19/52), the most common co-infected virus were influenza and respiratory syncytial virus. The main diagnoses of HMPV infection were pneumonia (29/52, 55.77%) and bronchitis (23/52, 44.23%), while the main clinical manifestations were cough, fever, rhinorrhoea, and sneeze. Among 48 HMPV-positive specimens, A2b (19/48, 39.58%) and B1 (26/48, 54.17%) were the main epidemic subtypes. Patients with HMPV genotype A infection had a higher viral load compared to genotype B patients (6.07 vs. 5.37 log_10_ RNA copies/ml). Five complete sequences of HMPV were obtained. This is the first report of a whole genome sequence of HMPV-B1 isolated in China.

**Conclusions:**

HMPV is an important respiratory pathogen in paediatric patients. Cases of HMPV infection could burden hospitals in the epidemic season. HMPV viral loads and genotypes have no correlation with co-infection or clinical characteristics.

## Background

*Human metapneumovirus* (HMPV) is an enveloped, non-segmented virus, with a negative-sense, single-stranded RNA genome, classified in the *Metapneumovirus* genus within the family *Pneumovirinae*. Since HMPV was first detected in 2001 [1], it has been determined to be one of the most common pathogens of acute respiratory tract infections (ARTIs) among all age groups, particularly affecting children, the elderly, and immunocompromised individuals [2, 3]. HMPV can cause both upper and lower respiratory tract infection (URTI and LRTI). The clinical symptoms of HMPV infection are similar to those of respiratory syncytial virus (RSV) infection, i.e. non-productive cough, fever, rhinorrhoea, and wheezing [2, 4].

The HMPV genome is about 13 Kb in length and is composed of eight genes encoding nine proteins: 3′-N, P, M, F, M2-1/M2-2, SH, G, L-5′. Based on gene sequences for the nucleoprotein (N), fusion protein (F), and glycoprotein (G), HMPV can be divided into five subtypes (A1, A2a, A2b, B1, and B2) [5, 6]. Some studies have identified other subtypes [7–9], such as A2c, B2a, and B2b, but the prevalence of these new subtypes requires support from more epidemiological data. At present, the correlation between HMPV genotypes and the severity of illness remains unclear.

In this study, we describe the prevalence rate, co-infection status, and clinical characteristics of HMPV in paediatric patients with ARTIs. Additionally, we explore the association between viral loads and symptoms of HMPV-infected patients and analyse the distribution of HMPV genotypes in Beijing, China. We also obtained five whole genome sequences of HMPV.

## Materials and methods

### Study population and specimen collection

From April 2017 to March 2018, nasopharyngeal aspirates (NPAs) were collected from children (aged < 14 years) with ARTIs who were hospitalized in the Beijing Friendship hospital. ARTIs were defined by the presence of at least two of the following symptoms: fever, cough, sneeze, wheezing, nasal obstruction, sore throat, and dyspnoea. Children who had been enrolled but discharged were considered as a new case if they were readmitted for a new episode of ARTIs. Demographic data and clinical characteristics were recorded on a form from each patient’s record of medical history and examination. All NPAs were collected in tubes with viral transport medium and kept at − 80 °C until use.

### Detection of HMPV

The viral nucleic acids were extracted from 200 µl of each specimen with a QIAamp MinElute Kit (Qiagen, Germany) in accordance with the manufacture’s protocols. HMPV was identified by using a One-step RT-PCR Kit (Ambion, USA) in accordance with the manufacture’s protocols. HMPV forward primer (5′-CATATAAGCATGCTATATTAAAAGAGTCTC-3′), reverse primer (5′-CCTATTTCTGCAGCATATTTGTAATCAG-3′), and probe (FAM-TGYAATGATGAGGGTGTCACTGCGGTTG-TAMRA) were used as previously described [10] to amplify a 163-bp fragment from the N gene. PCR-positive products were confirmed by sequencing. Viral loads were detected by real-time reverse transcription PCR (qRT-PCR), and the standard curve was generated as described previously [11].

### Detection of common respiratory viruses

HMPV-positive specimens were screened for 16 common respiratory viruses: influenza virus types A–C (IFV), parainfluenza virus types 1–4 (HPIV), human coronaviruses HKU1/229E/OC43/NL63 (HCoV), respiratory syncytial virus (RSV), human rhinovirus (HRV), human bocavirus (HBoV), adenovirus (ADV), and WU polyomavirus (WUPyV). All viruses were assayed by real-time PCR, and RNA viruses were tested using an AgPath-ID™ One-Step RT-PCR Kit (Ambion), DNA viruses were tested using TaqMan™ Gene Expression Master Mix (Thermo Fisher, USA).

### HMPV genotyping

HMPV cDNA was synthesized using SuperScript III First-Strand Synthesis System for Reverse Transcription-PCR (RT-PCR) (Invitrogen, USA) in accordance with the manufacture’s protocols. The partial sequence of N gene (1200 bp) was amplified using a nested PCR under the following thermocycling conditions: an initial denaturing at 94 °C for 5 min, followed by 35 cycles of 94 °C for 40 s, 46 °C for 40 s, and 72 °C for 1 min, and a final extension at 72 °C for 10 min. The following newly designed primers were used: outer forward primer, 5′-TTAARTTACAAAAAAACATGGGAC-3′; outer reverse primer, 5′-AAAGAATATCTTTTCCTTCAGGG-3′; internal forward primer, 5′-ATGGGACAAGTGAAAATGTCTC-3′; and internal reverse primer, 5′-AATTACTCATAATCATTTTGACTG-3′. Specimens that failed to be amplified were considered as untyped. The PCR products were sequenced by Sanger sequencing (TSINGKE, Beijing, China). A neighbour-joining (NJ) tree was constructed by the Tamura-Nei model in MEGA 7.0 using 1000 bootstrap replicates. The web server of FindModel (https://www.hiv.lanl.gov/content/sequence/findmodel/findmodel.html) was used to find best available nucleotide substitution model. Reference strains of HMPV were acquired from GenBank, including AF371337, AY297749, DQ843659, AY525843, and FJ168778. Avian metapneumovirus C (AMPV C, AY590688) was used as an outgroup to root the tree.

### Sequencing

HMPV-positive samples with high copy numbers (> 10^5^ copies/ml) were further used for whole genome sequencing. Fourteen pairs of primers with overlap were designed according to the reference strain CAN97-83 (GenBank accession number: AY297749) (Table 1). Ex-Taq (TaKaRa, China) was used to perform PCR. The PCR products were sequenced by Sanger sequencing, and the resulting sequences were assembled using Sequencher 5.0. Comparisons were made using published HMPV sequences selected from GenBank. A NJ-tree was constructed using the Tajima-Nei model (MEGA 7.0) with 1000 bootstrap replicates. Identity within the analysed sequences was analysed by BioEdit. Recombination events were detected by Simplot.

**Table 1:** Primer sets for amplification of the whole genome of HMPV

Target genes	Primers	Primer sequence (5′–3′)	Location (nt)^a^	Annealing temp (°C)
N	N(F)	GCGAAAAAAACGCGTATA	3–20	50
	N(R)	TTGCTGCTTCATTACCCAT	1296–1314	
P	P(F)	GAAGAAAAAGARGCTGCAGA	1178–1196	50
	P(R)	CCAGATCAACTTGAACAGC	2228–2246	
M	M(F)	GAATCMGAAGAAGAAGAAG	2070–2088	50
	M(R)	CAYCACTTTCCAAGACATT	3066–3084	
F	F-1(F)	CAATCMAAAAGGYATATTC	2800–2818	50.2
	F-1(R)	CTGCAGATGTYGGCATGT	3764–3781	
	F-2(F)	CAGTCAATTCAACAGAAG	3645–3662	50
	F-2(R)	TTCATATTTGCATGGAGC	4736–4753	
M2	M2(F)	CCTCCAGAGCTGARTGGTGT	4636–4655	50
	M2(R)	TWTCATTGTCAYTTATCCCA	5467–5486	
SH	SH(F)	GCAAGACAGTGAAAGCAYT	5254–5272	46.8
	SH(R)	CATCYTTGCTTTGARCAT	6268–6285	
G	G(F)	AAACAARAAWATGGGACAAG	6207–6226	50
	G(R)	TAGACATTRACAGTRGAYTC	7148–7167	
L	L-1(F)	GACAAATRRCAATGGATC	7122–7139	44.7
	L-1(R)	TAGTTCTGTYAARCTCTC	8303–8320	
	L-2(F)	GARAATGCTGCWGAATTA	8186–8203	46.8
	L-2(R)	GATATHGCYTCCATTGTCC	9288–9306	
	L-3(F)	ACATGCACCACCAGAAAC	9178–9195	46.8
	L-3(R)	AGGTGTTATGTTKTCWGC	10,238–10,225	
	L-4(F)	GTGAYATMAATAGAACAGC	10,128–10,146	43.8
	L-4(R)	ACTGAAGATRTGTTGATC	11,231–11,248	
	L-5(F)	GGAATTAGYATAATGAGTG	11,081–11,099	48
	L-5(R)	GGATATTCACATGCTGTTC	12,246–12,264	
	L-6(F)	CTAACAAGRAATTACATG	12,071–12,088	48
	L-6(R)	ACGGCAAAAAAACCGTAT	13,318–13,335	

^a^The reference of location is based on AY297749 strain

### Statistical analysis

Data analysis was performed using SPSS 13.0, and categorical data was compared by χ^2^ and Fisher’s exact tests. Two-tailed *p* values of < 0.05 were considered to indicate statistical significance.

## Results

### Epidemiology of HMPV

**Fig. 1:**
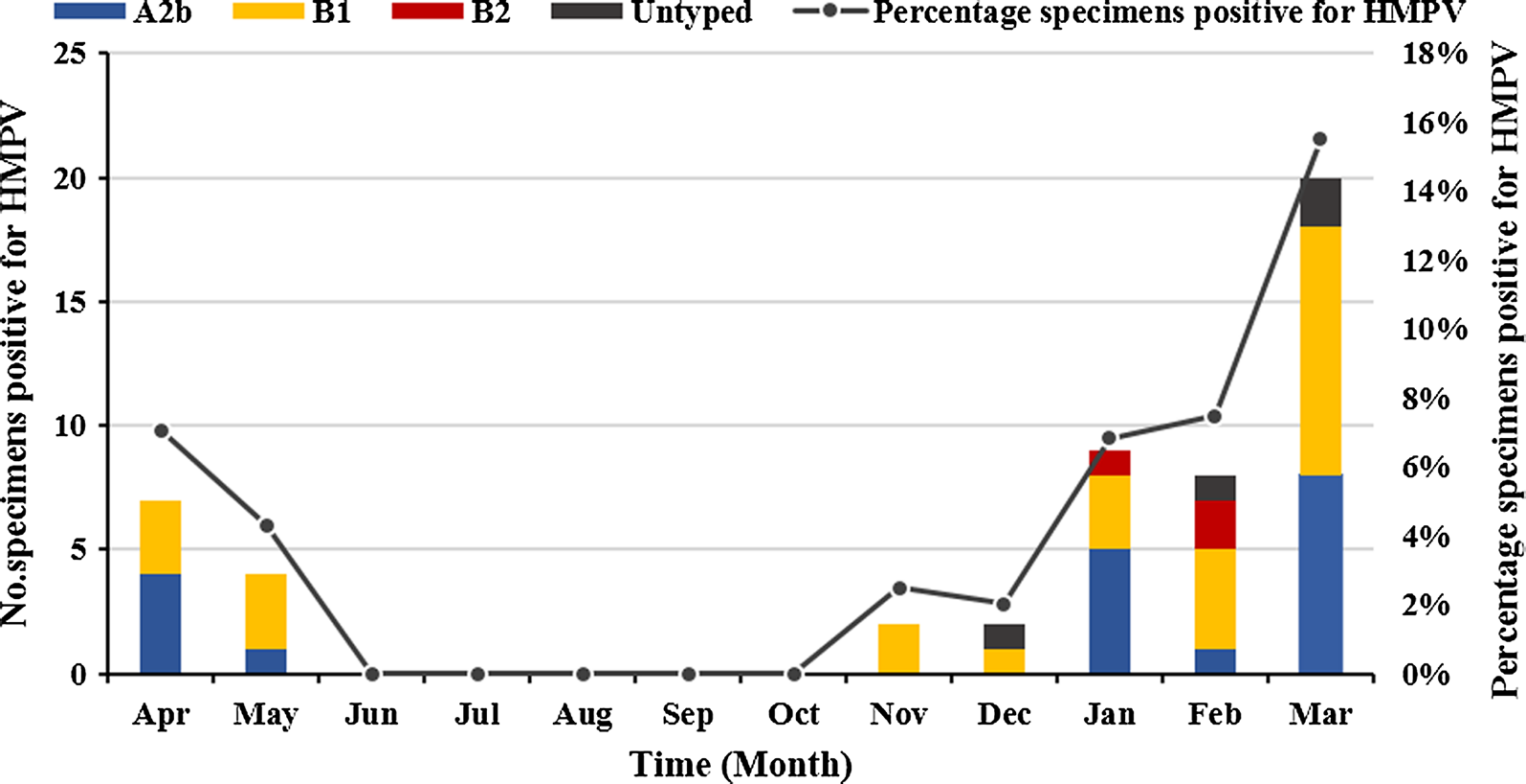
Seasonal distribution of HMPV-positive specimens from April 2017 and March 2018

A total of 1276 hospitalized children with ARTIs were enrolled between April 2017 and March 2018; 4.1% (52/1276) of their samples were positive for HMPV. As shown in Table 2, the male/female ratio was 1.26 (29:23) (*p* = 0.251), indicating the sex difference was not statistically significant. The median age of HMPV-infected children was 36 months (IQR: 1–168 months), and the detection rates were significantly different among different age groups (*p* = 0.019). The HMPV prevalence of patients aged ≤ 4 years (47/898, 5.2%) was significantly higher than that of patients aged 4–14 years (5/378, 1.3%) (*p* = 0.001). The seasonal distribution of HMPV infection from April 2017 to March 2018 is shown in Fig. 1. 59.6% (31/52) of positives were found in spring, none (0/52) in summer, 3.8% (2/52) in autumn and 36.5% (19/52) in winter. The detection frequencies peaked in March 2018 (20/129, 15.5%).

**Table 2:** Population demographic of HMPV-positive specimens from April 2017 and March 2018

Variable	Number of patient	Number of HMPV positive children	Prevalence of HMPV (%)	*p* value
*Gender*				0.251
Male	674	29	4.30	
Female	602	23	3.82	
*Age (years)*				0.019
≤ 1	340	13	3.82	
> 1 to ≤ 2	137	9	6.57	
> 2 to ≤ 3	281	17	6.05	
> 3 to ≤ 4	140	8	5.71	
> 4 to ≤ 5	86	0	0	
> 5 to ≤ 7	121	4	3.30	
> 7 to ≤ 14	171	1	0.58	
Total	1276	52	4.08

### Co-infection with other respiratory viruses

**Fig. 2:**
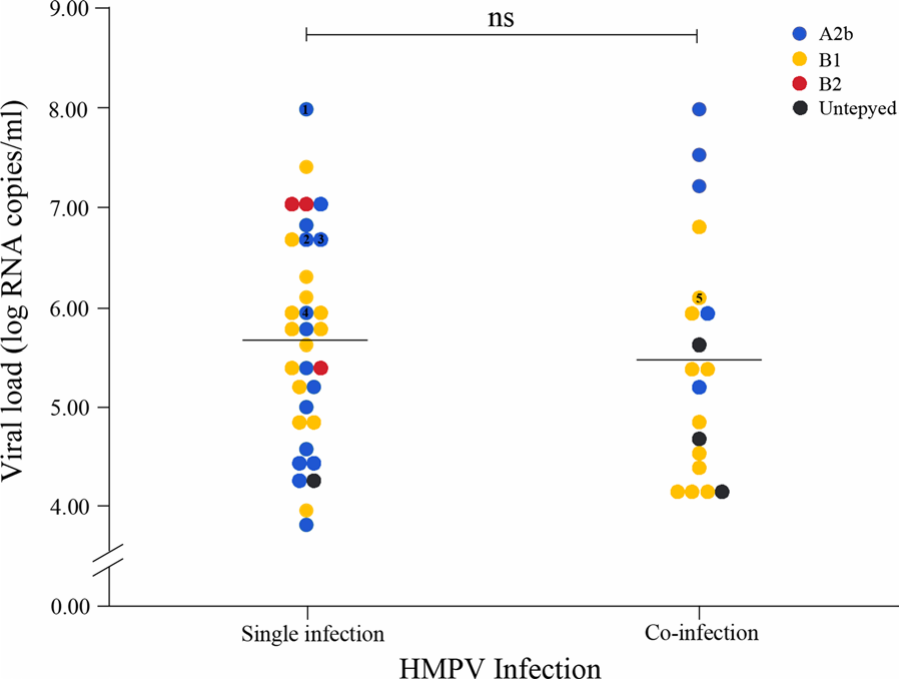
HMPV viral loads in NPAs from patients with or without co-infections. Numbers 1–5 represent samples from which the whole genome sequence is obtained

Among the 52 HMPV-infected children, 36.5% (19/52) were co-infected with other respiratory viruses (Table 3). Most co-infections involved RSV or IFV-A, (both account for 11.5%, 6/19). The linear range of the standard curve of the N-gene is 10^3^–10^12^ copies/ml. The viral loads of HMPV-positive patients ranged from 7 × 10^3^ to 9.61 × 10^7^ copies/ml. At the single time point when the viral load was measured, there was no statistical difference in the viral loads between HMPV mono-infections and co-infections (*p* = 0.398) (Fig. 2), and the viral loads had no relationship with the tested demographic or clinical features (i.e. gender, age, temperature, and hospitalization; data not shown).

**Table 3:** Co-detections of HMPV and other respiratory virus in the study

Groups	Co-infection viruses	NO. (%)
2 viruses (n = 14)	HMPV + IFV	4 (21.1)
	HMPV + RSV	3 (15.8)
	HMPV + HRV	2 (10.5)
	HMPV + WUPyV	2 (10.5)
	HMPV + ADV	1 (5.3)
	HMPV + HCoV	1 (5.3)
	HMPV + HBoV	1 (5.3)
3 viruses (n = 3)	HMPV + IFV + RSV	2 (10.5)
	HMPV + HRV + WUPyV	1 (5.3)
4 viruses (n = 1)	HMPV + HPIV + HCoV + ADV	1 (5.3)
5 viruses (n = 1)	HMPV + HPIV + RSV + ADV + WUPyV	1 (5.3)

*ADV* adenovirus, *HBoV* human bocavirus, *HCoV* human coronavirus (HKU1/229E/OC43/NL63), *HPIV* human parainfluenza virus 1–4, *HRV* human rhinovirus, *IFV* influenza virus A–C, *RSV* respiratory syncytial virus, *WUPyV* WU polyomavirus

### Clinical characteristics of HMPV infections

The clinical characteristics, diagnoses, and hospital stay lengths of HMPV-positive patients are listed in Table 4. The main clinical symptoms of HMPV infections included cough (98.1%, 51/52) and fever (temperature of ≥ 38 °C; 90.4%, 47/52); other common symptoms included rhinorrhoea (46.2%, 24/52), sneeze (26.9%, 14/52), shiver (21.2%, 11/52), and nasal obstruction (19.2%, 10/52). The duration of hospital stay for HMPV-infected children ranged from 4 to 18 days (mean: 6.75 ± 2.87 days), with most patients (78.9%, 41/52) being discharged within 7 days. Of the 52 HMPV-infected children, all were diagnosed with a LRTI, 94.2% (49/52) had an abnormal chest radiograph, 55.8% (29/52) had pneumonia, and 44.2% (23/52) had bronchitis. There was no significant difference in these symptoms between the HMPV-positive patients with co-infections and those without co-infections. Additionally, hospital stay, fever and cough have no significant difference between patients aged < 2 years and 2–14 years (*P* = 0.05, 0.187, 1.00, respectively).

**Table 4:** Clinical features among HMPV-positive hospitalized children

Clinical diagnoses and characteristics	Single infection NO. (%) (n = 33)	Co-infection NO. (%) (n = 19)	Total NO. (%) (n = 52)	*p*
Bronchitis	14 (42.4)	9 (47.4)	23 (44.2)	0.730^a^
Pneumonia	19 (57.6)	10 (52.6)	29 (55.8)	
Abnormal chest radiograph	31 (94.0)	18 (94.7)	49 (94.2)	1.000^b^
Hospitalization > 7d	5 (15.2)	6 (31.6)	11 (21.2)	0.296^b^
Fever (≥ 38℃)	29 (87.9)	18 (94.7)	47 (90.4)	0.641^c^
Convulsion	1 (3.0)	0 (0.0)	1 (1.9)	1.000^c^
Shiver	9 (27.3)	2 (10.5)	11 (21.2)	0.284^b^
Cough	33 (100.0)	18 (94.7)	51 (98.1)	0.365^c^
Nasal obstruction	7 (21.2)	3 (15.8)	10 (19.2)	0.910^b^
Rhinorrhoea	14 (42.4)	10 (52.6)	24 (46.2)	0.477^a^
Sneeze	8 (24.2)	6 (31.6)	14 (26.9)	0.566^a^
Vomit	7 (21.2)	1 (5.3)	8 (15.4)	0.256^b^
Diarrhea	1 (3.0)	1 (5.3)	2 (3.9)	1.000^c^

^a^χ2-test

^b^Continuity correction

^c^Fisher’s exact test

### Phylogenetic analysis of HMPV

**Fig. 3:**
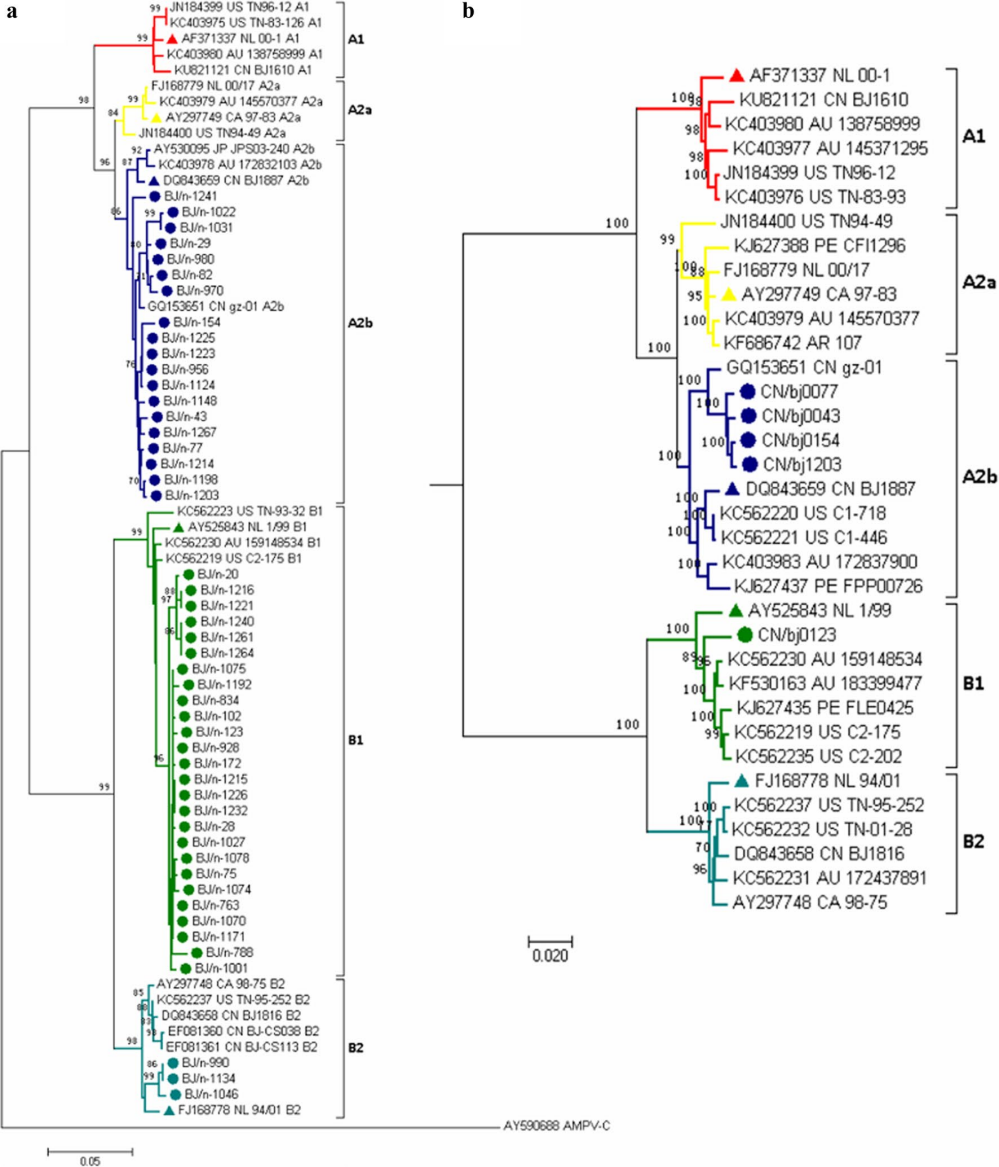
Phylogenetic relationships of the strains detected in HMPV-infected children. **a** NJ-tree constructed based on the partial N gene sequences (813 bp) of HMPV strains. **b** NJ-tree constructed based on the whole genome sequences of HMPV strains. The reference strain is marked with a ▲, and the strains analysed in this study are marked with ●. The country of origin for each reference and sample sequence is indicated by the following country codes: AR, Argentina; AU, Australia; CA, Canada; CN, China; JP, Japan; NL, Netherlands; PE, Peru; and US, United States of America

A portion of the N gene (813 bp) was amplified in 48 HMPV-positive specimens (the sequences are available in Additional file 1). The phylogenetic analysis conducted on these sequences revealed that 39.6% (19/48) of strains were grouped into the A2b lineage, 54.2% (26/48) of strains were grouped into the B1 lineage, and 6.3% (3/48) of strains were grouped into the B2 lineage; subtypes A1 and A2a were not detected (Fig. 3a). The positives rates of genotype A2b and B1 were higher in the spring (23.1%, 12/52 and 30.8%, 16/52, respectively). Additionally, HMPV genotype A patients had a significantly higher viral load (6.07 ± 1.21 log_10_ RNA copies/ml, n = 22) than genotype B patients (5.37 ± 0.92 log_10_ RNA copies/ml, n = 26) (*p* = 0.029). There was no statistical difference in the main clinical features (i.e. gender, age, temperature, hospitalization, and virus loads) between patients infected with HMPV genotype A and those infected with HMPV genotype B (data not shown).

### Whole genome analysis of HMPV

To characterize the whole genome sequences of HMPV epidemic strains, genomes were obtained for five of the HMPV strains by using Sanger sequencing; the GenBank accession numbers for these sequences are MK820375 and MN745084 to MN745087. Thirty HMPV sequences were downloaded from GenBank as reference strains. The Simplot analyses showed no evidence of a recombination event (data not shown). A phylogenetic tree based on the whole-genome sequences of HMPV was constructed using the NJ method. The resulting phylogenetic tree shows that four strains from this study belong to the A2b lineage, and are closest to the gz-01 (China) strain in genetic distance. Additionally, the first complete genome (bj0123) of an HMPV-B1 genotype in China was obtained. The results was consistent with the phylogenetic tree constructed based on the partial N gene sequences (Fig. 3b). Fusion protein (F) is an envelope protein of HMPV that has two functional sites. One is a cleavage site, Arg-Gln-Ser-Arg↓ (RQSR↓), which is important for fusion progress between HMPV and the host cell, and the other one is an Arg-Gly-Asp motif (RGD), which mediates the binding of virus and cellular *α*_v_*β*_1_ integrins [12, 13]. Based on an alignment of the obtained HMPV sequences with reference sequences, there were no nucleotide mutations detected in these functional sites (data not shown). The sequence identity were 80.0–100.0% among all the analysed sequences, G gene and SH gene have high genetic variability (48.3–100.0% and 66.4–100.0%, respectively). The identity among five obtained HMPV whole genomes were 80.1%–99.6%, while the identity of the four A2b genomes were 99.1–99.6%, and the identity between bj0123 and the four A2b genome is about 80.1%.

## Discussion

HMPV has been identified a leading cause of ARTIs since it was discovered in 2001. According to a serological study, HMPV infection has existed for at least 70 years [1]. Almost all children eventually become infected by HMPV, and adults can be re-infected by HMPV throughout their lives due to incomplete immunity. The prevalence of HMPV in children is approximately 2.0–18.2% [4, 14–16]. In this study, we explored the epidemiology and genotypic diversity of HMPV within hospitalized children in Beijing, China from April 2017 to March 2018. Of the 1276 children, 52 (4.1%) were positive for HMPV, while 2% in southern China (Guangzhou, 2014–2016) and 7.14% in northern China (Beijing, 2010) [4, 17], indicating that the prevalence of HMPV could be different in across regions and years. Therefore, it is necessary to establish continuous epidemiological surveillance in a wider area. The detection rates of HMPV infection between male and female patients in our study were not significantly different (*p* = 0.251), but there was a significance difference in the detection rates among age groups, with patients aged ≤ 4 years (4.8%) having a higher rate of detection compared with the patients aged 5–14 years (1.7%) (*p* = 0.001). Notably, 90.4% of HMPV infections detected in this study occurred in patients aged ≤ 4 years, which is consistent with findings from prior studies [4, 15]. The peak age range for HMPV infections was 1–2 years (6.6%).

HMPV infections have a clear tendency toward seasonal distribution, supported by the result of the present study (*p* = 0.000), but they vary based on different climate and geography factors. Here, HMPV was detected most frequently in the winter and spring. The HMPV detection rate was highest in March 2018, which is similar to findings from studies in Lanzhou (China) and Korea [18, 19].

Co-infection with respiratory viruses and bacteria is commonly seen in cases of ARTIs. Coinfections of HMPV with other respiratory viruses have been reported many times, and patients with such co-infections are more likely to have a fever, lead to severe pneumonia, and cause higher hospitalization rates [4, 18, 20]. The proportion of co-infection in this study was 36.5%. IFV and RSV were the most frequently co-infected viruses, which may be related to the overlap between the epidemic season of HMPV with those of IFV and RSV [21]. There was no significance difference observed between the co-infection group and mono-infection group in any of the assessed clinical manifestations, diagnoses, or hospital stay length. Peng et al. indicated that a high HMPV copy number is correlated with disease severity and hospital stay duration [22]; however, we did not find any relationship between the HMPV viral load and clinical features. There are also studies reporting that the viral load of asymptomatic children infected with HMPV is significantly lower than that of symptomatic children [23], which needs to be verified in future studies.

HMPV infection can cause both URTI and LRTI [3]. The risk factors associated with severe HMPV infection include preterm birth, early age, low immune function, nosocomial infection, and chronic pulmonary, cardiopathy, or neurological disease [24]. In the present study, all HMPV-infected patients had an URTI, 55.8% had pneumonia, and 44.2% had bronchitis. Additionally, 94.2% of HMPV-positive children had an abnormal chest radiograph, and the main clinical characteristics of HMPV infection were cough, fever, and rhinorrhoea. No severe cases were found in our cohort, and 78.9% of our patients were discharged within 7 days.

HMPV can be divided into genotypes A and B. Vicente et al. reported that type A HMPV is more virulent than type B, whereas Papenburg et al. concluded that type B HMPV was associated with severe infection [25, 26], and some studies found no evidence for differential severity among different HMPV genotypes [18]. In the present study, we found patients with HMPV genotype A infection had a higher viral load compared to genotype B patients (*p* = 0.029), which is consistent with Oong’s report [27]. However, there was no significant difference between HMPV genotypes A and B in terms of their epidemiological characteristics, hospital stay, or viral loads. We analysed the distribution of HMPV subtypes in Beijing based on a partial sequence of the N gene. Subtypes A2b, B1, and B2 were detected, but subtypes A1 and A2a were not found. The most prevalent subtype was B1 (54.5%), followed by A2b (40.9%), which is consistent with previous work conducted in Beijing, Changsha, Kuala Lumpur, and Jordanian [7, 14, 28, 29].

Finally, to study the genome variation of HMPV epidemic strains in Beijing, we obtained five complete HMPV genome sequences with 80.1–99.6% identity, and four of them have an identity of 99.1–99.6%. Phylogenetic tree results show that four strains belong to sublineage A2b and have the closest genetic distance to the gz-01 (China) strain, one strain belongs to sublineage B1, which is the first report of whole genome sequence of HMPV-B1 isolated in China. No recombination events or mutations in important function sites were found.

The study is limited by the lack of a control group without ARTIs and its short research duration. Follow-up studies with long-term continuous monitoring that include a control group are needed to analyse the clinical features more accurately.

## Conclusion

HMPV is an important virus in paediatric patients. The findings provide characteristics about the epidemiological and genotypic diversity of HMPV infections in Beijing, which will help to provide a theoretical basis for the prevention and control of diseases and to enrich the HMPV genome database of China.

## Supplementary Information

**Additional file 1.** The fasta data of the 48 N gene sequences.

## Data Availability

Condensed anonymized data are available from the corresponding author on reasonable request.

## Abbreviations

ADV: Adenovirus
AMPV: Avian metapneumovirus
ARTIs: Acute respiratory tract infections
HBoV: Human bocavirus
HCoV: Human coronavirus
HMPV: Human metapneumovirus
HPIVs: Human parainfluenza viruses
HRV: Human rhinovirus
IFV-A: Influenza A
LRTI: Lower respiratory tract infection
MEGA: Molecular evolutionary genetics analysis
NJ: Neighbour-joining method
NPAs: Nasopharyngeal aspirates
qRT-PCR: Real-time reverse transcription PCR
RNA: Ribonucleic acid
RSV: Respiratory syncytial virus
RT-PCR: Reverse transcription polymerase chain reaction
SPSS: Statistical product and service solutions
URTI: Upper respiratory tract infection
WUPyV: WU polyomavirus
